# Whole-genome sequence data of a *Salmonella enterica* serovar *Rissen* sequence type 8877 isolated from cracked table egg in Sudan

**DOI:** 10.1016/j.dib.2023.109005

**Published:** 2023-02-23

**Authors:** Mortada M.O. Elhassan, Habiba B.A. Adam, Shaimaa S.A.O. Fagir, Sofia B. Mohamed, Mona A.M. Khaier, Romisa Abdulaziz, Arshad Ismail, Mushal Allam

**Affiliations:** a College of Veterinary Medicine, University of Bahri, Khartoum, Sudan; b National University Biomedical Research Institute National University, Khartoum, Sudan; c College of Medicine and Health Sciences, United Arab Emirates University, Al Ain, United Arab Emirates; d National Institute for Communicable Diseases, National Health Laboratory Service, Johannesburg, South Africa

**Keywords:** *Rissen* ST8877, *Salmonella*, Sudan

## Abstract

*Salmonella enterica* serovar *Rissen* is an emerging and important *Salmonella* serovar prevalent in live animals and foods from retail markets worldwide. Here, we describe the whole-genome sequence of *Salmonella enterica* Serovar *Rissen* Sequence Type 8877 isolated from a cracked table egg in Sudan. The whole-genome sequencing was obtained using Illumina Miseq platform. The quality of the sequenced read, the De novo assembly, and the sequencing typing was conducted by JEKESA pipeline (https://github.com/stanikae/jekesa). The assembled genome was also uploaded to the Center for Genomic Epidemiology web server to determine acquired antibiotic resistance genes, predict the serovar, and the antigenic profile. The genome of *Salmonella enterica* serovar *Rissen* 1-M1 was found to harbor 4,689 protein-coding genes, 96 RNA genes, and 115 pseudogenes, as predicted by NCBI Prokaryotic Genome Annotation Pipeline. This whole genome shotgun project has been deposited at DDBJ/ENA/GenBank under accession JAPSFB000000000.

**Specifications Table**

**Table:** 

Subject	Microbiology

Specific subject area	Pathogen genomics
Type of data	Genome sequence data
How the data were acquired	Illumina MiSeq
Data format	Raw, filtered, and assembled genome
Description of data collection	*Salmonella enterica* Serovar *Rissen* was isolated from a cracked table egg using Xylose Lysine Deoxycholate medium. Bacterial DNA was extracted from a pure colony of *Salmonella* using DNA QIAamp DNA Mini Kit (Qiagen). The genome sequence was obtained by Illumina Miseq platform.
Data source location	• *Institution: College of Veterinary Medicine, University of Bahri* • *City/Town/Region: Khartoum* • *Country: Sudan*
Data accessibility	Repository name: GenBank (at National Center for Biotechnology Information, NCBI) Data identification number: JAPSFB000000000.1 Direct URL to data: https://www.ncbi.nlm.nih.gov/nuccore/JAPSFB000000000.1

**Value of the Data**

• The whole genome sequence data can be used successfully to evaluate the genetic diversity of *Salmonella enterica* serovar *Rissen*.
• Bioinformaticians can use the genome data in comparative genome analysis as well as evolution *of Salmonella enterica* serovar *Rissen*.
• The data may also help to investigate the genomic epidemiology of this pathogen.

## Objective

Chicken-derived products, particularly table eggs, are the most common source of salmonellosis, one of the most difficult diseases to control in the poultry production industry. Several outbreaks of salmonellosis have been reported, in which the eggs are the source of human infection; especially in the case of undercooked or raw eggs [1,2]. Table eggs can be contaminated on the outer shell surface and internally by any species or serovars of *Salmonella* [3]. Here, we present a whole-genome sequence analysis of a newly described sequence type of *Salmonella enterica* serovar *Rissen* ST8877 isolated from a cracked table egg obtained from a local market in Khartoum, Sudan. To our knowledge, this is the third detection of *Rissen* ST8877 [4], [5], [6], however, this is the first description of its whole genome.

## 1 Data Description

Table 1 shows the genome features of *Salmonella enterica* serovar *Rissen* sequence type 8877. The sequencing of isolate 1-M1 yielded 2089,086 raw reads. The high-quality reads (2038,422 reads) were assembled to 55 contigs (the longest contig was 521,231 bp) covering 4875,799 bp, with a GC content of 52.1% and *N*_50_ value of 237,445 bp. The genome of 1-M1 was found to harbor 4689 protein-coding genes, 96 RNA genes, and 115 pseudogenes, as predicted by the NCBI PGAP. MLST based on the *Salmonella enterica* seven-allele scheme indicated ST8877. ResFinder detected aminoglycoside resistance gene *aac(6′)-Iaa* (98.1%, NC_003197) which increases resistance to tobramycin and/or amikacin [7]. Serotyping of 1-M1 resulted in the serovar *Rissen* and the antigenic profile was identified as O7:H1f.g:H2- (H2 antigen was not identified). PlasmidFinder identified only Col (pHAD28) plasmid replicon sequence (91.6 %, KU674895). Up to 23 SPIs were detected in these genomes, including SPI-1 (7 islands), SPI-2 (8 islands), SPI-3 (2 islands), SPI-4 (1 island), SPI-5 (1 island), SPI-8 (1 island), SPI-9 (1 island), C63PI (1 islands), and CS54 (1 island). The Whole Genome Shotgun project has been deposited at DDBJ/ENA/GenBank under the accession JAPSFB000000000. The version described in this report is version JAPSFB010000000.

**Table 1:** The genome characteristics of *Salmonella enterica* serovar *Rissen* sequence type 8877 isolated from a cracked table egg.

Item	Value
GenBank accession number	JAPSFB000000000.1
NCBI BioSample No.	SAMN32036406
Number of reads	2,086,086
Number of contigs	55
GC content (%)	52.1
N50 (bp)	237,445
Coverage (×)	100X
Total length (bp)	4,875,799
Total No. of CDS	4689
Serovar	*Rissen*
Sequence type	ST8877
AMR genes	aac(6′)-Iaa (Aminoglycoside)
SPI	SPI-1 (7), SPI-2 (8), SPI-3 (2), SPI-4 (1), SPI-5 (1), SPI-8 (1), SPI-9 (1), C63PI (1), and CS54 (1)
CRISPR Arrays	2

## 2 Experimental Design, Materials and Methods

In 2019, a cracked egg (produced by a white Leghorn commercial laying chicken) was collected from a retail shop in a local market in Khartoum city in Sudan. The egg was collected under aseptic conditions and analyzed immediately within 2 hours using previously described methods for isolation and identification [8]. The egg content was mixed thoroughly before taking a sample for bacteriology. A sterile swab was soaked in normal saline and then applied to the surface of the egg. The swab was then dipped into a test tube containing 10 ml normal saline and the contents were mixed thoroughly using a vortex mixer. Serial dilutions up to 10^5^ were then prepared. One ml was taken from appropriate dilution and inoculated into Xylose-Lysine Deoxycholate agar (M031, HiMedia, India), a proper selective medium for the isolation of *Salmonella* [9]. The culture was incubated at 37 °C for 2–48 h and examined for the presence of typical colonies of *Salmonella* (red with a black centre). The colonies were further subjected to biochemical testing. The DNA was extracted using the QIAamp DNA minikit (Qiagen, Germany), and paired-end libraries were prepared using the Nextera DNA Flex library kit, followed by 2  ×  300 bp sequencing on a MiSeq machine (Illumina, Inc., USA). For whole-genome sequence analysis and typing, the JEKESA pipeline (https://github.com/stanikae/jekesa) was used. Briefly, Trim Galore v0.6.2 (https://github.com/FelixKrueger/TrimGalore) was used to filter the sequence reads (Q, ≥ 20; length, ≥ 50), de novo assembly was performed using SPAdes v3.13.2 (https://github.com/ablab/spades),the assemblies were polished and optimized using Shovill v1.1.0 (https://github.com/tseemann/shovill), and sequence typing was done using the multilocus sequence typing (MLST) tool v2.16.4 (https://github.com/tseemann/mlst). Assembly metrics, including the GC content and number of contigs, were calculated using QUAST v5.0.2 (http://quast.sourceforge.net/quast). All resultant contiguous sequences were annotated using the NCBI Prokaryotic Genome Annotation Pipeline v4.13 [10]. Furthermore, the assembled genome was uploaded to the Center for Genomic Epidemiology web server; to determine acquired antibiotic resistance genes using ResFinder v4.1 [11], [12], [13]; to predict the serovar and the antigenic profile (O, H1 and H2 antigens) using SeqSero v1.2 [14], to identify plasmids using PlasmidFinder v2.1 [13,15], and to determine salmonella pathogenicity islands using SPIFinder v2.0 [16].

## Ethics Statements

This work did not involve studies with animals or humans.

## Credit Author Statement

**Mortada M. O. Elhassan**: Conceptualization, design of the research, and the analysis of the results; **Habiba B. A. Adam**: Collection of samples and bacteriology work; **Shaimaa S. A. O. Fagir**: Bacteriology work; Sofia B. Mohamed and Mona A. M. Khaier: DNA extraction; **Romisa Abdulaziz**: Analysis of the results; **Arshad Ismail**: Whole genome sequencing; **Mushal Allam**: Analysis of the results, writing and editing.

## Declaration of Competing Interest

The authors declare that they have no known competing financial interests or personal relationships that could have appeared to influence the work reported in this paper.

## Data Availability

Salmonella enterica subsp. enterica serovar Rissen strain 1-M1, whole genome shotgun sequencing project (Original data) (GenBank).
